# Exercising and Compression Mechanism in the Treatment of Lymphedema

**DOI:** 10.7759/cureus.16121

**Published:** 2021-07-02

**Authors:** Stelamarys Barufi, Henrique Jose Pereira de Godoy, Jose Maria Pereira de Godoy, Maria de Fatima Guerreiro Godoy

**Affiliations:** 1 Physiotherapy, Faculdade de Medicina de São José do Rio Preto (FAMERP) Member Research Group of the Clínica Godoy, São José do Rio Preto, BRA; 2 General Surgery, Faculdade de Medicina de São José do Rio Preto (FAMERP) Member Research Group of the Clínica Godoy, São José do Rio Preto, BRA; 3 Cardiology and Cardiovascular Surgery, Faculdade de Medicina de São José do Rio Preto (FAMERP), São José do Rio Preto, BRA; 4 Angiology and Vascular Surgery, Clínica Godoy, São José do Rio Preto, BRA; 5 Postgraduate Health Science Course in Medicine School of São José do Rio Preto (FAMERP) Coordinator Group of Rehabilitation Department in Clinica Godoy, Faculdade de Medicina de São José do Rio Preto (FAMERP) Clínica Godoy, São José do Rio Preto, BRA

**Keywords:** lymphedema, active exercising, compression garment

## Abstract

Aim: The aim of this study was to evaluate the effect of adjustments to a compression stocking on reductions in leg volume during walking in patients with lymphedema.

Method: Fourteen women and three men suffering from leg lymphedema with ages between 21 and 68 years old (mean 45.68 years) were randomly enrolled in this study. Evaluations were made by volumetry before and after each session of controlled walking. Patients were subjected to three one-hour sessions of walking slowly on the flat ground monitored by a professional. For the first session, the patients used a well-adjusted cotton-polyester compression stocking, for the second they used a badly adjusted compression stocking made of the same fabric, and for the third, no compression garment was used. The Kruskal-Wallis test was used for statistical analysis.

Results: On comparing the volume before and after walking for one hour with the well-adjusted cotton-polyester compression stocking, there was a mean reduction of 46.2 mL ± 66.95 mL (p-value < 0.02) in the volume of the lymphedema. In the one-hour session of walking without any compression, the volume of the leg increased by 74.4 mL ± 99.75 mL (p-value < 0.007). On walking with the compression stocking badly adjusted, there was a mean increase in the volume of 31.6 mL ± 46.9 mL (p-value < 0.14).

Conclusion: Walking is a type of muscle activity that can be transformed into a type of daily exercise when you are guided by how it is going to be performed. The exercise, in this study specifically, walking, with a strict control of speed and time of realization using a compression mechanism is well adjusted to the volume of the limb and surely effective in reducing edema.

## Introduction

Lymphedema affects many populations, there is no cure, and there are few therapeutic prospects involving, in general, the private sector. This situation is aggravated in low-income countries where a lack of government resources and specialized health workers has led to the marginalization of the disease [1].

There is no consensus on a single therapy to treat lymphedema; however, manual lymph drainage [2], compression therapy [3], exercising [4], and hygienic care [5] constitute the cornerstones of treatment. Recently, other options such as mechanical lymph drainage with devices that use both passive [6] and active [7] muscle movements, psychological support [8], and myolymphokinetic activities [9] have improved the treatment in these patients. Myolymphokinetic activities are day-to-day activities that involve the mobility of the limb and can be transformed into a form of treatment. Several studies, both ongoing and in press, show that the association of these activities with compression garments maintains losses achieved during lymph drainage [10-12].

 Walking is a type of muscle activity that can be transformed into a type of daily exercise when you are guided by how it is going to be performed. A pilot study of the Godoy & Godoy poster presented at the 24th World Congress of Lymphology, Italy, September 2013, suggested that when compared the volume before and after walking for one hour with the well-adjusted cotton-polyester compression stocking show reductions in the volume of leg lymphedema. The aim of this study was to evaluate the effect of adjustments to a compression stocking on reductions in leg volume during walking in patients with lymphedema.

## Materials and methods

Method

Seventeen patients presented with a diagnosis of lower limb bilateral below the knee, congenital primary bilateral, and early primary lymphedema. All initial diagnoses were performed more than five years ago and patients were selected in a cross-over study where all patients already participated in the rehabilitation group and were selected on a first-come basis at the Clinica Godoy-São José do Rio Preto-Brazil. The group was made up of 14 women and three men with ages between 21 and 68 years and a mean age of 45.68 years.

 The inclusion criterion was the presence of any type of Grade II leg lymphedema diagnosed clinically or by lymphoscintigraphy. The exclusion criteria were lymphedema associated with osteoarticular problems and active infections identified during the initial evaluation and other clinically diagnosed causes of edema.

 An assessment was made by water-displacement volumetry (following the principle of Archimedes) before and immediately after each session of walking utilizing a measuring container especially developed for this examination.

 All patients were submitted to three evaluations using mechanism compression made grosgrain fabric, custom made according to the size of the limb, use eyelets to allow a certain amount of adjustment directly by the patient. For one session the patients walked slowly for one hour using a well-adjusted cotton-polyester (grosgrain) compression stocking, without any ‘space’ between the compression stocking and the skin, for the second they used a badly adjusted cotton-polyester compression stocking (that was loose and so there was ‘space’ between it and the skin), and for the third, no cotton-polyester compression stocking was used. The stockings were tailor-made for each patient.

 The compression device used in this study was a stocking made from a cotton-polyester fabric called grosgrain. Grosgrain is a fabric that has important features including a low stretch and yet ribbed structure. The ribbed aspect improves the fit around the leg compared to other textiles. Additionally, the fabric stretches < 50% of its size along the direction of the ribs but stretches more across the ribs thereby facilitating joint flexibility without losing compression.

 The participants performed the sessions in no specific order (cross-over). The patients always walked on flat ground and in the morning between 8:00 and 9:00 o’clock and were monitored by a physiotherapist who controlled the speed at which they walked. The speed of walking was according to the physical condition of each patient. The patients were asked to walk at their normal speed without stopping.

 The Kruskal-Wallis test, Dwass-Steel-Critchlow-Fligner, and paired t-tests were used for statistical analysis with an alpha error of 5% being considered acceptable. Before starting the study, all participants signed informed consent forms. This study was approved by the Research Ethics Committee of the Medicine School in São José do Rio Preto (#374/2009).

## Results

There was a mean reduction of 46.2 mL (± 66.95 mL, 1.2% reduction of volume; paired t-test p-value < 0.02) in volume while walking with a well-adjusted compression garment.

 The volume increased by an average of 74.5 mL (± 99.75 mL, 1.9% increase of volume; paired t-test p-value < 0.007) while walking without any compression.

**Figure 1:**
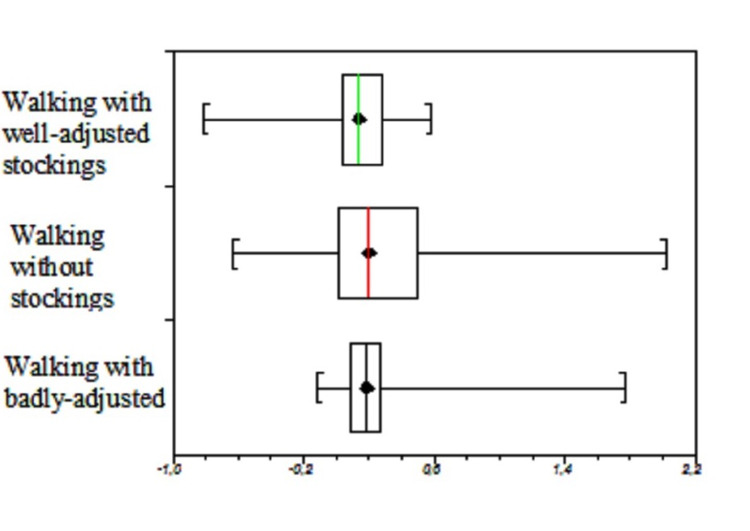
Variations in the total volume of the lymphedematous leg before and after walking using grosgrain well-adjusted stockings, without stockings, and badly-adjusted stockings.

 Although insignificant, there was a mean increase in the volume of 31.6 mL (± 46.9 mL, 0.8% increased of volume; paired t-test; p-value < 0.14) during the walking session using a badly adjusted cotton-polyester compression garment. Figure 1 shows the median variations and the quartiles of the three evaluations. The Kruskal-Wallis test identified significant differences between the three groups and the pairwise comparisons (Dwass-Steel-Critchlow-Fligner) identified significant differences between the changes in leg volumes while walking with the well-adjusted stockings and both with badly adjusted and without stockings. Moreover, there was no significant difference between the use of badly adjusted stockings and no stockings (Table 1 and Figure 1).

**Table 1:** Pairwise comparisons (Kruskal-Wallis and Dwass-Steel-Critchlow-Fligner) for the three evaluations. A, well adjusted; B, badly adjusted; WO, no stockings; *p-value ≤ 0.05

Difference A vs. Difference B	p-value = 0.0096*
Difference A vs. Difference WO	p-value = 0.0002*
Difference B vs. Difference WO	p-value = 0.5232

## Discussion

This study shows that walking is a type of muscle activity that can be transformed into a type of daily exercise when you are guided by how it is going to be performed. When we associated mechanism compression a synergistic effect occurs in reducing the volume of lymphedema, that is, the association of two mechanisms enhances the reduction in the volume of edema. The compression garment used in this study is made from a cotton-polyester fabric called grosgrain; this fabric is perfect to exert low-stretch compression of up to 50% [13]. While walking without any compression stocking, there was an increase in the volume of the lymphedematous leg. In the evaluation with badly adjusted compression, there was also an increase, albeit insignificant, in the size of the leg. However, when the compression stocking was well-adjusted, there was a significant reduction in the volume of the leg, thereby proving that the adjustment of the compression garment has a synergistic effect in reducing the volume of lymphedematous legs.

This assessment is educationally important for patients and even for professionals, as it proves the importance of careful adjustments to maximize the result. The necessity of constant adjustments in nonelastic and low-stretch compression lies in the mechanism of action; muscle activity generates varying working pressures on the interface between the skin and the compression device. Thus, the hypothesis is that the reduction in the size of the leg leads to a decrease in the effectiveness of the changes in working pressure to provide reductions until at one point this pressure no longer exists. From this moment, further losses in volume will not occur and the effectiveness of the treatment is lost; this explains the need for continuous adjustments of the compression garment.

These stockings are different from elastic stockings because the latter have both resting and working therapeutic pressures [13]. Hence, for nonelastic compression devices to work, the compression garment must be well adjusted. These devices, with activity, provide a continuous reduction in the edema and require that this reduction is followed by adjustments in the size of the compression garment.

Another aspect to be considered is that when muscles work, they require a higher blood flow and therefore greater capillary filtration and an increased need for lymphovenous drainage; a reduction in edema of the limb depends on this balance. If muscle activity requires a larger amount of blood than the capacity of lymphovenous drainage, the edema will increase.

This study shows that when patients with lymphedema walked without any type of compression device, the lymphedema worsened; when the patients walked with a badly adjusted compression device, the edema increased slightly, but when the compression was well adjusted, the edema reduced. In this study, the muscle activity was controlled and the only variable that changed was the type of compression. However, the type of muscular activity must be controlled to assess the balance between capillary filtration and lymphovenous drainage.

Thus in the treatment of lymphedema, it is essential to maintain the trophism and the integrity of the venous return including the muscle propulsion and aspiration pumps [14]. Failure of the venous system overloads the lymphatic system, the functional reserve of the venous system, thereby aggravating lymphedema. Therefore, this integrity becomes paramount. In this case, the muscle activity of patients with lymphedema becomes part of the treatment [15]. Hence, as long as muscle activity does not aggravate lymphedema, it should be recommended. Other important aspects to be considered in exercising are the effect of gravitational pressure, the integrity of the joints, and the possibility of using intensive forms of treatment [16].

Elastic stockings on patients with chronic venous disease may also help to reduce the size of the legs while walking [15]. However, for the treatment of lymphedema, nonelastic or low-stretch compression is suggested. It is recommended to wear a compression garment the entire day (24 hours) until there is no more lymphedema.

One suggestion is the development of devices to evaluate the resting and working pressures that are routinely experienced with compression therapy. Thus, it will be possible to identify the best time to replace stockings by checking that they are well adjusted and to maintain the efficacy of compression therapy. The limitation of the current study is that it could have been carried out in other types of lymphedema as a secondary, thus we would be able to evaluate how this type of exercise behaves in this type of lymphedema. However, this study is already an objective of the researchers.

## Conclusions

Walking is a type of muscle activity that can be transformed into a type of daily exercise when you are guided by how it is going to be performed. The exercise, in this study specifically, walking, with a strict control of speed and time of realization using a compression mechanism is well adjusted to the volume of the limb and surely effective in reducing edema.
